# Preoperative antibiotic prophylaxis and the incidence of surgical site infections in elective clean soft tissue surgery of the hand and upper limb: a systematic review and meta-analysis

**DOI:** 10.1186/s10195-024-00748-4

**Published:** 2024-01-28

**Authors:** Gabrielle Avelar Negri, Antônio Clodoildo Andrade Junior, Manuela Amoedo Cox, Marcos Felipe Marcatto de Abreu, Simone Appenzeller, Rodrigo Gonçalves Pagnano

**Affiliations:** 1 Department of Orthopedics, Rheumatology and Traumatology-School of Medical Science, University of Campinas (Unicamp), 126 Tessália Vieira de Camargo St, Cidade Universitária, Campinas, SP 13083-887 Brazil; 2 Campinas, Brazil

**Keywords:** Antibiotic prophylaxis, Premedication, Upper extremity, Postoperative complications, Surgical wound infection, General surgery

## Abstract

**Background:**

Surgical site infections (SSI) are the most frequent early complications of hand surgeries. However, the indications still remain uncertain for antibiotic prophylaxis in elective clean soft tissue surgeries of the hand and upper limb. Therefore, a systematic review of the literature and a meta-analysis was conducted to investigate the impact of antibiotic prophylaxis on the prevention of SSI in these types of surgeries.

**Methods:**

An electronic search was performed in the following databases: MEDLINE/Pubmed, PMC/Pubmed, Web of Science/Clarivate Analytics, Embase/Elsevier, Scopus/Elsevier, BVS/Lilacs, and the Cochrane Library, with no restrictions regarding publication language or date. The primary outcome of interest was the occurrence of SSI following elective clean soft tissue surgeries of the hand and upper limb according to the administration of preoperative antibiotic prophylaxis and no antibiotic prophylaxis. Surgeries involving simultaneous bone procedures or orthopedic implants were excluded. Study selection and data extraction were conducted independently by two reviewers. RoB 2.0 and ROBINS-I are Cochrane risk-of-bias tool for randomized trials and non-randomized studies of interventions. The magnitude of the intervention effect was estimated using the relative risk (RR). The meta-analysis was performed with the Review Manager and R software tools, using the Mantel–Haenszel random-effects model and a 95% confidence interval (CI). Results with *p* ≤ 0.05 were considered statistically significant. The quality of evidence was assessed using the Grading of Recommendations, Assessment, Development, and Evaluation (GRADE) approach.

**Results:**

The initial search yielded 1175 titles, from which 12 articles met the inclusion criteria for the systematic review, and 10 were included in the subsequent meta-analysis. The majority of these studies were nonrandomized intervention trials, exhibiting a moderate risk of bias. According to our review, preoperative antibiotic prophylaxis did not have a statistically significant impact on the incidence of SSI (RR = 1.13, 95% CI 0.91–1.40, *p* = 0.28). The overall quality of evidence for this outcome was rated as low. Moderate statistical heterogeneity was observed (*I*^2^ = 44%), and the prespecified sensitivity analysis highlighted the consistency of the results.

**Conclusions:**

While these results were consistent with the findings from individual studies included in this review, it is important to note that, given the threshold of *p* ≤ 0.05 for statistical significance, no definitive conclusions can be drawn from the quantitative analysis of the data obtained.

*Level of evidence*: Level 2.

*Trial registration*: CRD42023417786.

**Supplementary Information:**

The online version contains supplementary material available at 10.1186/s10195-024-00748-4.

## Introduction

Surgical site infections (SSI) are the most frequent early complications of elective soft tissue hand surgeries. Despite millions of these surgical procedures being performed each year, these infections are rare, with rates between 0.3% and 1.5%, and are predominantly superficial [1–4].

Measures to control and prevent these outcomes include, among others, antibiotic prophylaxis. There is evidentiary support for the use of preoperative antibiotic prophylaxis for many orthopedic procedures (e.g., open fractures, lower-extremity fractures, and total joint replacement), but not for elective soft tissue hand surgical procedures [1, 3].

In this context, a recent international study with members of the American Society of Surgeons of the Hand showed that around half of the surgeons did not prescribe prophylactic antibiotics for the surgical treatment of carpal tunnel syndrome [5]. Likewise, an interview with members of the British Society for Surgery of the Hand found that around 80% of surgeons did not prescribe them for the surgical treatment of Dupuytren’s disease [6]. Prior to surgery, 13.6% (2009e2015) of patients received prophylactic intravenous antibiotics and trend analysis showed a statistically significant increase from 2009 (10.6%) to 2015 (18.3%), an increase of 72.5% [7].

However, observational, nonrandomized, nonblinded, single-center studies and, mainly, with a statistical power compromised by a sample size that is not large enough and representative of the investigated effect, have prevented the development of specific guidelines for careful antibiotic prophylaxis in these surgeries [8, 9]. As a result, decisions about the administration of preoperative antibiotic prophylaxis in elective clean soft tissue surgeries of the hand and upper limb are still based on the institution’s traditions and the surgeon’s preferences [1, 5, 6, 9, 10].

Therefore, this study aimed to investigate, through a systematic literature review and a meta-analysis, the impact of preoperative antibiotic prophylaxis on the prevention of SSI in this class of surgeries.

## Methods

This review was conducted according to the Preferred Reporting Items for Systematic Reviews and Meta-analyses (PRISMA) statement, published in 2020, as demonstrated in additional file 1 (“PRISMA 2020 Checklist”) [11]. The study question was developed using the PICO acronym, where “P” represents the study population (patients submitted to elective clean soft tissue surgeries of the hand and upper limb), “I” defines the intervention to be investigated (administration of preoperative prophylactic antibiotics), “C” refers to the comparison of treatments (administration of placebo or no antibiotic prophylaxis), and “O” refers to the outcome investigated (occurrence of SSI). Therefore, the study question was: “Does preoperative antibiotic prophylaxis in elective clean soft tissue surgeries of the hand and upper limb prevent SSI?.

A systematic electronic search was performed in April 2023 in the following databases: MEDLINE/Pubmed, PMC/Pubmed, Web of Science/Clarivate Analytics, Embase/Elsevier, Scopus/Elsevier, BVS/Lilacs, and the Cochrane Library, using the search strategy that was built and validated with the collaboration of a librarian from the School of Medical Sciences at UNICAMP: (“Antibiotic Prophylaxis” OR Premedication) AND ((Hand AND “Upper Extremity”) OR Hand) AND “General Surgery” AND (“Postoperative Complications” OR “Surgical Wound Infection”), as described in additional file 2 (“Search strategies and information sources”). As “soft tissue” did not enable to retrieve relevant articles in preliminary searches, this term was discarded. The study protocol is available in the Prospective Register of Systematic Reviews (PROSPERO) international database under code CRD42023417786.

Our review included articles published in any period, language, or country, whose human adult or pediatric patients had undergone elective clean soft tissue surgeries of the hand and upper limb and whose primary outcome—incidence of SSI after this class of surgeries—had been described with the administration of preoperative antibiotic prophylaxis (any antimicrobial or dosage used) and without antibiotic prophylaxis.

The following articles were excluded: (1) studies that did not discriminate soft tissue surgeries from those involving simultaneous bone procedures or orthopedic implants, or clean surgeries from those in which patients had history of previous local infection; (2) studies whose postoperative follow-up was less than 4 weeks; (3) studies performed with animals or in vitro studies; (4) literature reviews, systematic reviews and meta-analyses, case series and reports, book chapters, letters, expert comments or opinions, expert panel, consensus statements, editorials, interviews, seminars, posters; and (5) unpublished or incomplete articles or articles that did not provide enough data to define the eligible population or assess the primary outcome.

Study selection and data extraction were conducted independently by two reviewers (GAN, MAC), according to predefined eligibility criteria. Disagreements were resolved by consensus among reviewers or arbitration by a senior reviewer (MFMA).

Selected articles had their full texts revised and data extracted into an especially developed form containing the variables of interest: main author, year of publication, country, conflict of interests, funding sources, study design, follow-up, surgical procedures performed, demographic characteristics of the population and potential risk factors for SSI, sample size, absolute number of participants who met the eligibility criteria of this systematic review, absolute number of patients who received preoperative antibiotic prophylaxis (case group), absolute number of patients who received placebo or no drug prophylaxis (control group), absolute number of cases that evolved to SSI in each group, absolute number of cases with severe surgical site infections, i.e., that required a new surgical approach or hospitalization for infection treatment, occurrence of minor complications of wounds, occurrence of adverse reactions and side effects related to antimicrobials, and information about costs when comparing interventions.

Two independent reviewers (GAN, ACAJ) assessed the risk of bias using the Cochrane tools RoB 2.0 and ROBINS-I for randomized trials and nonrandomized intervention studies, respectively [12, 13]. Finally, the quality of evidence was classified according to the Grading of Recommendations, Assessment, Development, and Evaluation (GRADE) approach.

### Meta-analysis

Statistical analyses were performed using the Review Manager and R software tools. The magnitude of the intervention effect was estimated by relative risk (RR) and the Mantel–Haenszel random-effects model was used in the meta-analysis. A 95% confidence interval (CI) was adopted and *p* ≤ 0.05 was considered statistically significant. Heterogeneity was assessed through visual inspection of forest plots and Cochran’s *Q*-tests (*p* ≤ 0.1), *I*^2^, and Tau^2^, and the recognition of significant heterogeneity would lead to verification of collected data, exclusion of relevant outliers, and primary studies with inconsistent methodological characteristics, as well as comparison of results obtained using meta-analyses of fixed and random effects. Finally, publication bias was assessed by visual inspection of the funnel plot and Egger’s test (*p* ≤ 0.05).

## Results

### Review statistics

**Fig. 1:**
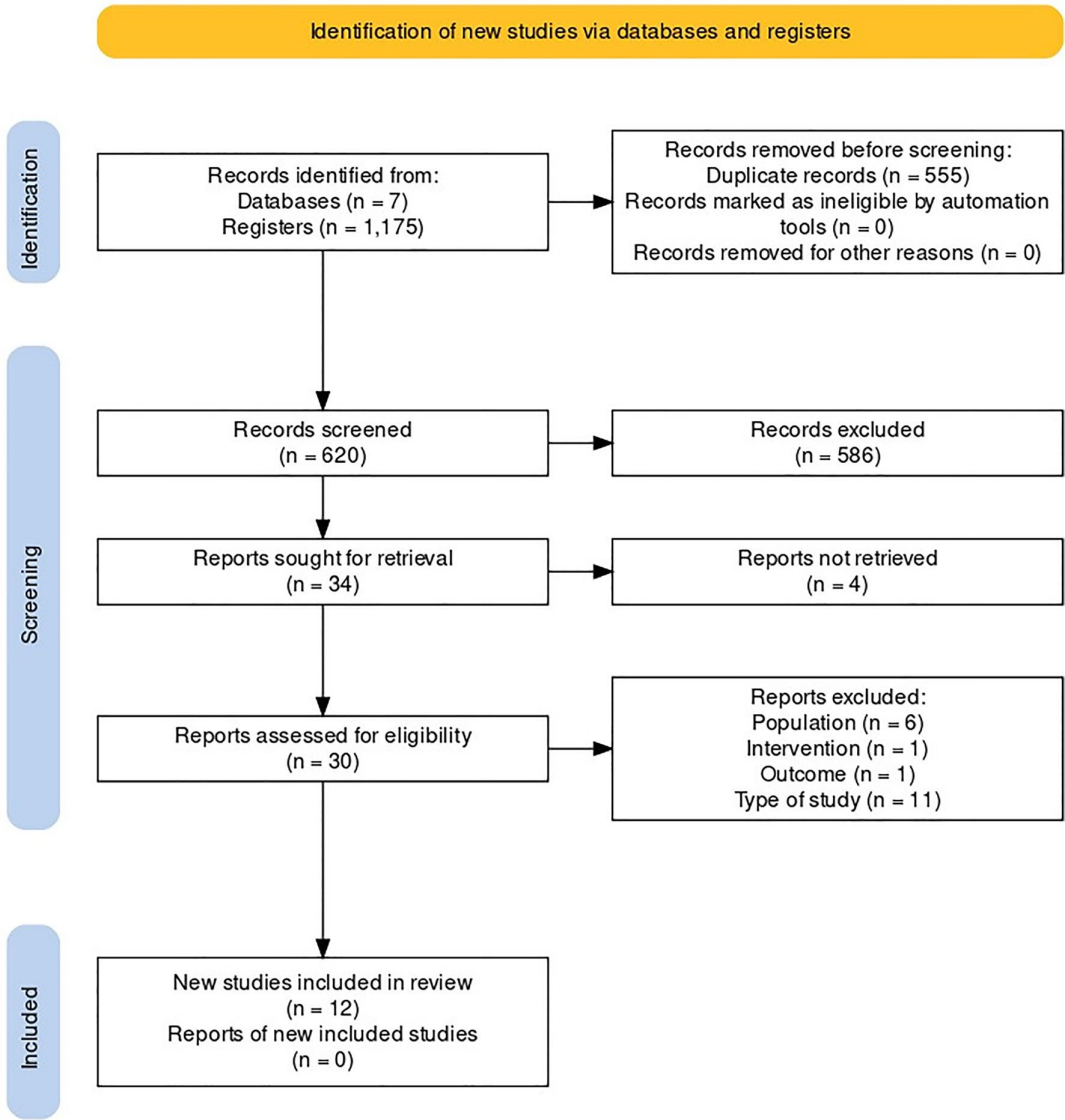
PRISMA flow diagram

The initial search strategy found 1175 articles; of these, 555 were removed due to duplicates. After reading this titles and abstracts, 44 were selected for full-text review. Of these, 18 were excluded for not meeting the eligibility criteria and 4 because they were not fully available, as detailed in additional file 3 (“Reports excluded”). Therefore, 12 articles were included in this systematic review and 10 in the meta-analysis, two of which were excluded (Hoel et al. and Wachtel et al.) for presenting a number of events equal to zero in at least one of the comparison groups [4, 7, 14–23] (Fig. 1).

### Study quality assessment

Eligible articles addressed the most common elective clean soft tissue surgeries of the hand and upper limb in clinical practice (e.g., open and endoscopic carpal tunnel release and carpal tunnel release revision, trigger finger release, cubital tunnel release, ulnar nerve transposition at the elbow, release of the ulnar nerve at the wrist, first extensor compartment release, fasciectomy for palmar fibromatosis and Dupuytren contracture release, tumor excision, tenosynovectomy of flexor tendons, fasciotomy, soft tissue laceration, tendon injury, nerve injury and/or vessel injury, tendon transfer, and wrist soft tissue arthroscopy) and had a combined population sample of 817,805 participants. Cefazolin was the antimicrobial used in prophylaxis of most studies, except in cases of previous reactions to cephalosporin or penicillin, when clindamycin was used instead [7, 14, 15, 17, 20–23]. The minimum postoperative follow-up time was 30 days in all studies and the diagnostic criteria can be considered more or less homogeneous [4, 7, 16–23]. Tables 1 and 2 summarize the main characteristics of the primary studies included in our review and each of the comparison groups.

**Table 1:** Summary of articles included in this systematic review

	Main author and year	Country	Study design	Follow-up (days)	Surgical procedures	Demographic characteristics	Number of participants
1	Aydin N (2010) [14]	Turkey	Single-center clinical, randomized, double-blind, placebo-controlled trial	180	Patients underwent surgeries for contracture, soft tissue laceration, carpal tunnel release and fasciotomy, fasciectomy for palmar fibromatosis, tumor excision, tendon injury, nerve injury and/or vessel injury, and tendon transfer	Mean age 30.92 ± 2.4 years Sex (M/F) 74.4%/25.6% This study excluded patients with immunosuppression and other comorbidities, such as diabetes and smoking	1340 Total number of participants who meet the eligibility criteria for this systematic review: 426
2	Bykowski MR (2011) [16]	USA	Single-center retrospective observational study	79	Patients underwent surgeries for carpal tunnel release (*n* = 3783), trigger finger release (*n* = 1992), first extensor compartment release (*n* = 1046), wrist cyst excision (*n* = 625), excision of subfascial tumor in the hand (*n* = 421), release of the ulnar nerve at the wrist (*n* = 392), ulnar nerve transposition at the elbow (*n* = 358), excision of subcutaneous tumor of the hand (*n* = 201), excision of subcutaneous tumor of the forearm and wrist (*n* = 32) Mean surgery time: 27 ± 21 min		8,850
3	Bäcker HC (2021) [15]	USA	Multicenter, prospective nonrandomized clinical study	42	Patients underwent open carpal tunnel release (*n* = 143), revision of carpal tunnel release (*n* = 2), endoscopic carpal tunnel release (*n* = 54), trigger finger release (*n* = 134), first extensor compartment release (*n* = 26), fasciectomy for palmar fibromatosis (*n* = 16), cubital tunnel release (*n* = 1), tenosynovectomy of flexor tendons (*n* = 3), excision of tumors (*n* = 102), foreign body excision (*n* = 1), nail biopsy (*n* = 1), implant removal (*n* = 1)	Mean age 61 years Sex (M/F) 38.5%/61.5%	434
4	Harness NG (2010) [4]	USA	Multicenter, retrospective observational study	30	Surgeries performed: open and endoscopic carpal tunnel release and carpal tunnel release revision		2336
5	Hoel RJ (2018) [17]	USA	Single-center, retrospective observational study	30	Surgeries performed were wrist arthroscopy; 116 patients (36%) underwent concomitant open procedures, 6 also involved the use of skin closure material and belonged to the group that received preoperative antibiotics; therefore, they were excluded from data analysis and from this systematic review	Diabetes mellitus 3% Smoking 13%	324 Total number of participants who meet the eligibility criteria for this systematic review: 318
6	Johnson SP (2018) [7]	USA	Multicenter, retrospective observational study assessing databases of medical claims	30	Surgeries analyzed: open carpal tunnel release (*n* = 209,275), endoscopic carpal tunnel release (*n* = 48,282), trigger finger release (*n* = 4490), first extensor compartment release (*n* = 20,598), removal of tumor at the wrist (*n* = 23,301)	Sex (M/F) 35%/65% Diabetes mellitus 25.2% Charlson comorbidity index 0–52.4%/1% to 3–8.3% 4% to 8–22%/ > 8%–17,3%	305,946 Total number of participants who meet the eligibility criteria for this systematic review: 285,642
7	Li K (2018) [18]	USA	Multicenter, retrospective observational study assessing databases of medical claims	30	Patients underwent surgeries of carpal tunnel release (*n* = 250,613), trigger finger release (*n* = 119,390), first extensor compartment release (*n* = 25,972), removal of tumor at the wrist (*n* = 121,011)	Sex (M/F) 64%/36%	516,986
8	Mehta S (2022) [19]	USA	Single-center, retrospective observational study	30	Patients underwent surgeries of carpal tunnel release	Mean age 59 ± 14 years Sex (M/F) 35.2%/64.8% IMC 34 ± 9.4 kg/m^2^ Diabetes mellitus 26.6% Smoking 12.7% HbA1c > 7 10% ESRD 1.7%	770
9	Tosti R (2012) [20]	USA	Multicenter, retrospective observational study	30	Patients underwent surgeries of carpal tunnel release (*n* = 300), trigger finger release (*n* = 175), first extensor compartment release (*n* = 44), removal of tumor at the wrist (*n* = 81)	Diabetes mellitus 23.8% Smoking 20.8%	600
10	Vasconcelos C (2017) [21]	Portugal	Single-center, retrospective observational study	30	Patients underwent surgeries of carpal tunnel release (*n* = 211), trigger finger release (*n* = 68), first extensor compartment release (*n* = 61), removal of tumor at the wrist (*n* = 6) Mean surgery time: 19.6 min, with all eligible participants undergoing procedures with surgery time of less than 30 min	Mean age 58.4 years Sex (M/F) 14.4%/85.6% Comorbidities 70.8% Diabetes mellitus 14.4%	346
11	Wachtel N (2023) [22]	Germany	Multicenter, ambispective observational study	30	Patients underwent wrist soft tissue arthroscopy. Most arthroscopy procedures performed involved pathology of the triangular fibrocartilage complex (30.9%), the other procedures included arthroscopic resection of cysts (23.6%), purely diagnostic arthroscopy with or without synovectomy (18.5%), combined procedures (19.1%), procedures involving pathologies of the intrinsic ligaments of the wrist (5.1%), among others, such as loose body removal (2.8%) Mean surgery time: 40.4 ± 18.1 min	Mean age 38.1 years Sex (M/F) 42.7%/57.3% Comorbidities 61.2% Diabetes mellitus 1.7% Smoking 24.7% Alcohol consumption 18% IT 5.6% Prior SSI 2.2%	178; of these, 56.2% were recruited retrospectively and 43.8% were recruited prospectively
12	Zheng A (2022) [23]	USA	Single-center, retrospective observational study	30	Patients underwent surgeries of cubital tunnel release	Mean age 54.6 (12–87) years Sex (M/F) 52.7%/47.3%	919

*M* male, *F* female, *BMI* body mass index, *HbA1c* glycosylated hemoglobin, *SSI* surgical site infection, *ESRD* end-stage renal disease, *IT* immunosuppressive therapy

**Table 2:** Summary of articles included in the systematic review: intervention and control group

Main author and year		Intervention group				Control group			
		Intervention: preoperative antibiotic prophylaxis	Outcome: SSI	Demographic characteristics	Comorbidities	Intervention: placebo or no drug prophylaxis	Outcome: SSI	Demographic characteristics	Comorbidities
1	Aydin N (2010) [14]	211 (cefazolin)	8			215 (placebo)	7		
2	Bykowski MR (2011) [16]	2.755	15	Mean age 55 ± 15 years Sex (M/F) 36.2%/63.8% BMI 29.1 ± 6 kg/m^2^	Smoking 14.9% Diabetes mellitus 12.8%	6095 (none)	16	Mean age 52 ± 15 years Sex (M/F) 37.3%/62.7% BMI 28.6 ± 6 kg/m^2^	Smoking 19.1% Diabetes mellitus 10%
3	Bäcker HC (2021) [15]	177 (cefazolin)	1	Mean age 63.4 years Sex (M/F) 36.7%/63.3%	Comorbidities 14.1%	257 (none)	1	Mean age 58.5 years Sex (M/F) 39.7%/60.3%	Comorbidities 23.7%
4	Harness NG (2010) [4]	1.419	5	Median age 56 (48–66) years Sex (M/F) 29.7%/70.3%	Diabetes mellitus 12.7%	917 (none)	6	Median age 57 (49–69) years Sex (M/F) 34.1%/65.9%	Diabetes mellitus 26.6%
5	Hoel RJ (2018) [17]	203 (cefazolin/clindamycin)	0, because the 2 cases of infection underwent percutaneous fixation with Kirschner wires			115 (none)	0		
6	Johnson SP (2018) [7]	37.741 (cefazolin, vancomycin, gentamicin, among others)	140			247,901 (none)	710		
7	Li K (2018) [18]	58.201	832	Mean age 53 ± 15 years Sex (M/F) 37.1%/62.9%	Diabetes mellitus 20.7% Smoking 11.9% RA 3%	458,785 (none)	6933	Mean age 54 ± 15 years Sex (M/F) 35.9%/64.1%	Diabetes mellitus 19% Smoking 6.3% RA 2.7%
8	Mehta S (2022) [19]	491	19	Mean age 59 ± 14 years Sex (M/F) 34.6%/65.4% BMI 34 ± 9.7 kg/m^2^	Diabetes mellitus 29.5% Smoking 13.4% HbA1c > 7 12.6% ESRD 2.2%	279 (none)	9	Mean age 58 ± 14 years Sex (M/F) 36.2%/63.8% BMI 34 ± 8.9 kg/m^2^	Diabetes mellitus 21.5% Smoking 11.5% HbA1c > 7 5.4% ESRD 0.7%
9	Tosti R (2012) [20]	212 (cefazolin/vancomycin or clindamycin)	1	Mean age 52 ± 14.9 years Sex (M/F) 31.6%/68.4%	Diabetes mellitus 27.3% Smoking 23.1%	388 (none)	3	Mean age 55.9 ± 14.7 years Sex (M/F) 32.2%/67.8%	Diabetes mellitus 22% Smoking 17%
10	Vasconcelos C (2017) [21]	180 (cefazolin)	2	Mean age 58.4 years Sex (M/F) 13.3%/86.7%	Comorbidities 21.1% Diabetes mellitus 15%	166 (none)	2	Mean age 58.5 years Sex (M/F) 15.7%/84.3%	Comorbidities 62% Diabetes mellitus 13.8%
11	Wachtel N (2023) [22]	69; of these, 54 patients were recruited retrospectively and 15 prospectively (cefuroxime or clindamycin)	0	Mean age 38.6 years BMI 25.4 kg/m^2^	Comorbidities 24.1% Diabetes mellitus 0 Smoking 27.5% Alcohol consumption 21.7% IT 5.8% Prior SSI 2.9%	109; of these, 46 patients were recruited retrospectively and 63 prospectively (none)	0	Mean age 37.8 years BMI 24.7 kg/m^2^	Comorbidities 60.5% Diabetes mellitus 2.7% Smoking 23% Alcohol consumption 24.7% IT 5.5% SSI 18.3%
12	Zheng A (2022) [23]	623 (cefazolin, clindamycin, among others)	17			296 (none)	7		

*M* male, *F* female, *BMI* body mass index, *HbA1c* glycosylated hemoglobin, *SSI* surgical site infection, *ESRD* end-stage renal disease, *IT* immunosuppressive therapy, *RA* rheumatoid arthritis

A rate of 0.3–3.64% of SSI was observed after this class of surgeries in selected primary studies, and no statistical difference was observed in the incidence of infections with the administration of preoperative antibiotic prophylaxis [4, 7, 16–23]. Tables 3 and 4 show serious complications resulting from SSI, such as required surgical re-treatment and/or hospitalization, and possible adverse reactions and side effects related to the use of antimicrobials.

**Table 3:** Serious complications secondary to SSI

Main author and year	Total number of eligible participants	Case group (ATB)		Control group (PL/∅)		Serious complications secondary to SSI
		Total number	Events (SSI)	Total number	Events (SSI)	
Bykowski MR (2011) [16]	8850	2755	15	6095	16	Eight patients required a new surgical approach to treat SSI
Bäcker HC (2021) [15]	434	177	1	257	1	One patient required a new surgical approach to treat SSI
Harness NG (2010) [4]	2336	1419	5	917	6	Ten patients required a new surgical approach to treat SSI; of these, one required two surgical procedures for cleaning and debridement; one case was considered deep infection (organ/cavity) in the case group and three in the control group
Mehta S (2022) [19]	770	491	19	279	9	None
Tosti R (2012) [20]	600	212	1	388	3	None
Vasconcelos C (2017) [21]	346	180	2	166	2	None
Zheng A (2022) [23]	919	623	17	296	7	Five patients required a new surgical approach and/or hospitalization to treat SSI, three in the case group and two in the control group
*Total*	14,255	5857	60	8398	44	24

**Table 4:** Adverse reactions and side effects to antimicrobials

Main author and year	Total number of eligible participants	Case group (ATB)		Control group (PL/∅)		Adverse reactions and side effects to antimicrobials
		Total number	Events (AR/SE)	Total number	Events (AR/SE)	
Wachtel N (2023) [22]	178	69	Abdominal pain (*n* = 3) Meteorism (*n* = 5) Diarrhea (*n* = 3) Nausea (*n* = 4) Eczema (*n* = 2)	109	Abdominal pain (*n* = 1) Meteorism (*n* = 2) Diarrhea (*n* = 0) Nausea (*n* = 4) Eczema (*n* = 1)	To identify cases of adverse reactions and side effects to antimicrobials, patients were asked about the occurrence of signs or symptoms of intestinal disorders (meteorism, abdominal pain, nausea or vomiting, and diarrhea) and hypersensitivity reactions (eczema, pruritus, and anaphylactic shock) in the first 14 days after arthroscopy, which were classified by the patients as mild or severe. Patients who received preoperative antibiotics had significantly more adverse reactions and side effects related to the administration of these drugs than patients who did not receive antibiotic prophylaxis (16.2% versus 5.5%; *p* = 0.029, chi-squared test). Also, one out of ten patients who use antimicrobials has adverse reactions and side effects to these drugs

**Fig. 2:**
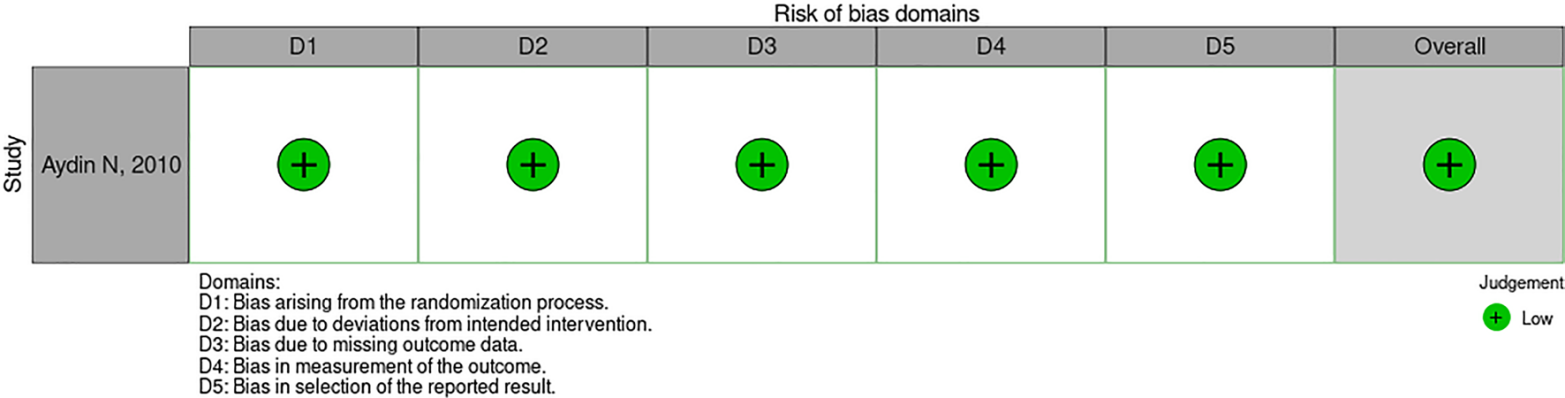
Traffic light chart: risk of bias in randomized clinical trials (RoB 2.0)

**Fig. 3:**
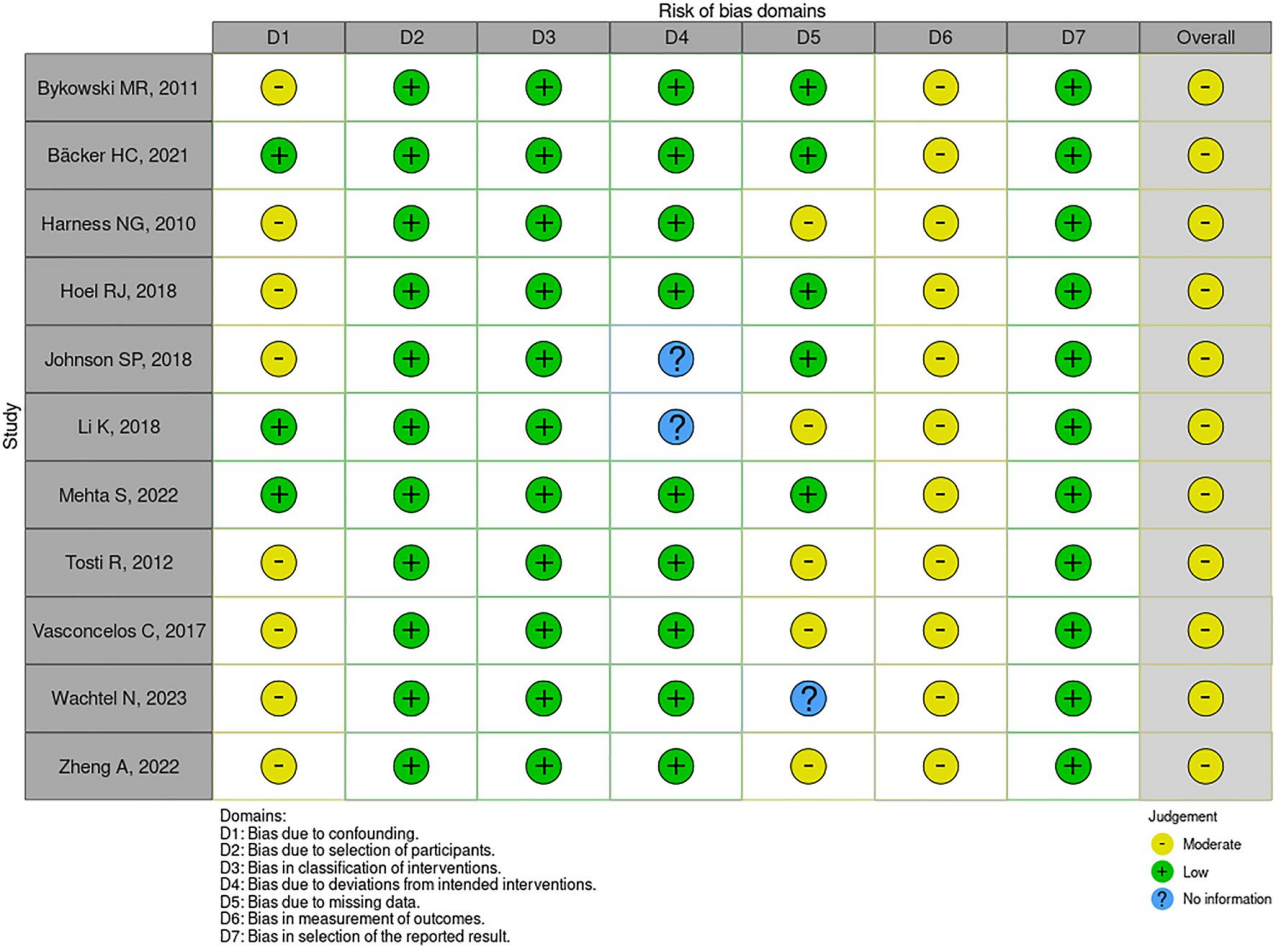
Traffic light chart: risk of bias in nonrandomized intervention studies (ROBIS-I)

**Fig. 4:**
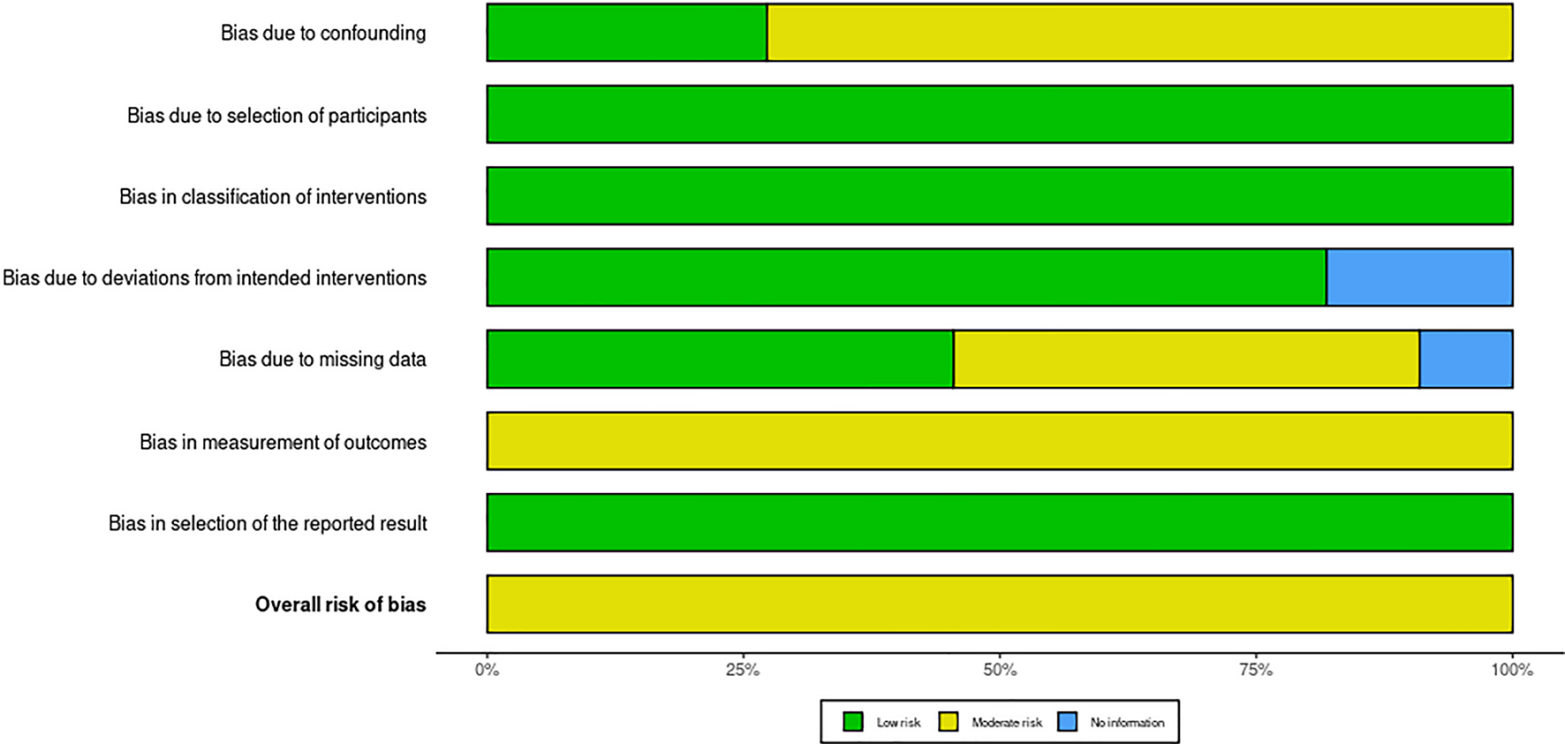
Weighted bar chart: risk of bias in nonrandomized intervention studies (ROBIS-I)

One of these studies was a randomized double-blind, placebo-controlled clinical trial, with low risk of bias in RoB 2.0, as illustrated in Fig. 2 [14]. Another one was a prospective intervention study whose patients were categorized into groups according to the institution where they were admitted, observing a moderate risk of bias in ROBIS-I mainly due to the lack of secrecy regarding the intervention, with possible bias in outcome evaluation [15]. The other studies included were observational cohort studies, all of them presenting a moderate risk of bias in the ROBIS-I, as illustrated in Figs. 3 and 4 [4, 7, 16–23]. Of these, the studies by Johnson et al. and Li et al. accounted for 98.14% of participants in our systematic review and were conducted using databases of medical claims and, therefore, have methodological limitations attributed to reliance on adequate coding and lack of access to medical records and patients [7, 18].

Tosti et al. did not declare any conflicts of interest while conducting their study [20]. Harness et al. received funding from the Kaiser Foundation Health Plan and Zheng et al. from the National Institutes of Health [4, 23]. The authors of the other articles declared no potential conflicts of interest regarding their studies, authorship, and/or article publication, and that no funding was received for their studies, authorship, and/or article publication. [4, 7, 14–23]

### Meta-analysis

**Fig. 5:**
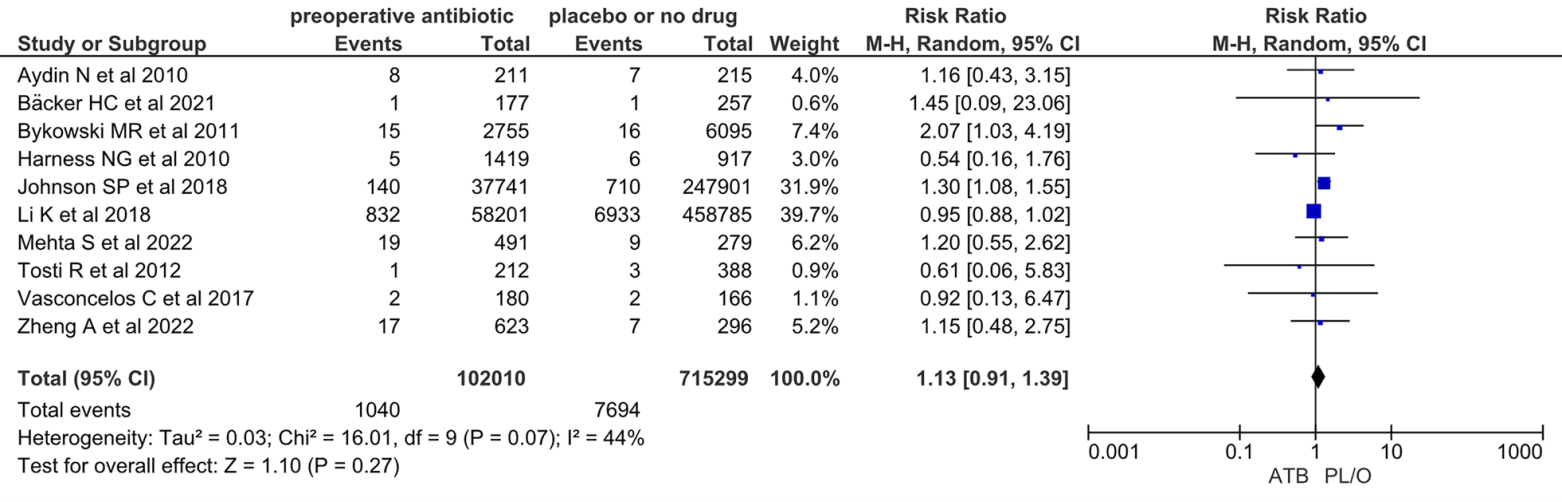
Forest plot: Mantel–Haenszel random-effects model (Review Manager software)

In the quantitative data analysis, the prescription of preoperative prophylactic antibiotics did not have a statistically significant effect on the prevention of SSI when compared with the administration of placebo or no antibiotic prophylaxis (RR = 1.13; 95% CI 0.91–1.39; *Z* = 1.1; *p* = 0.27) (Fig. 5).

**Fig. 6:**
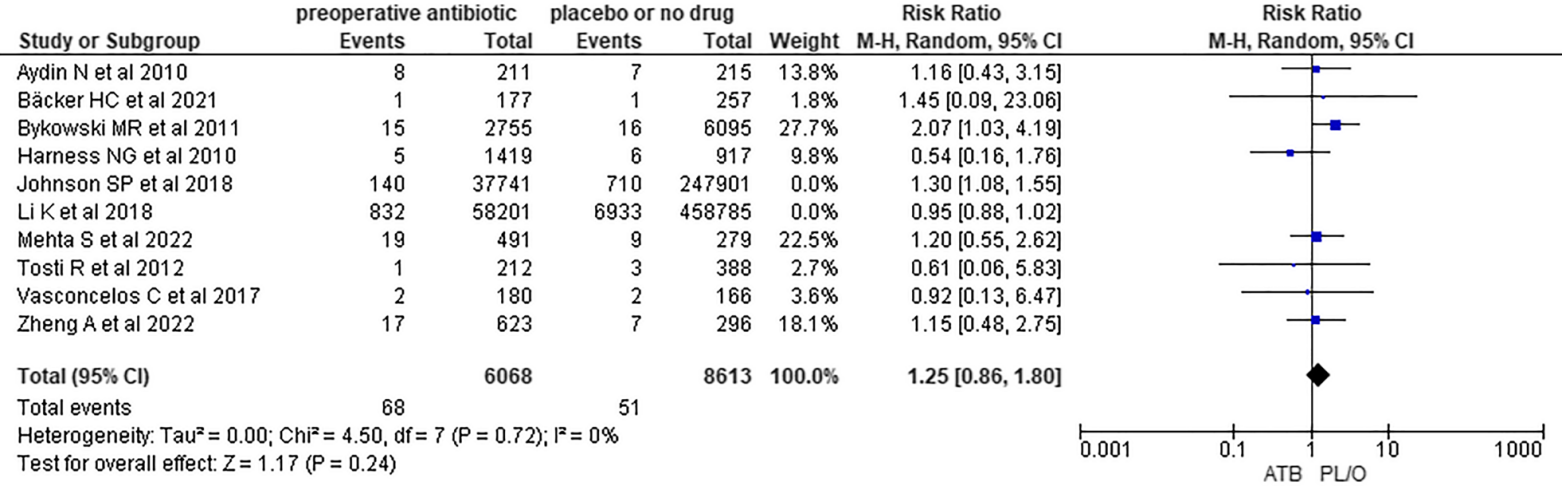
Sensitivity analysis: exclusion of studies performed in databases of medical claims [6, 7]

**Fig. 7:**
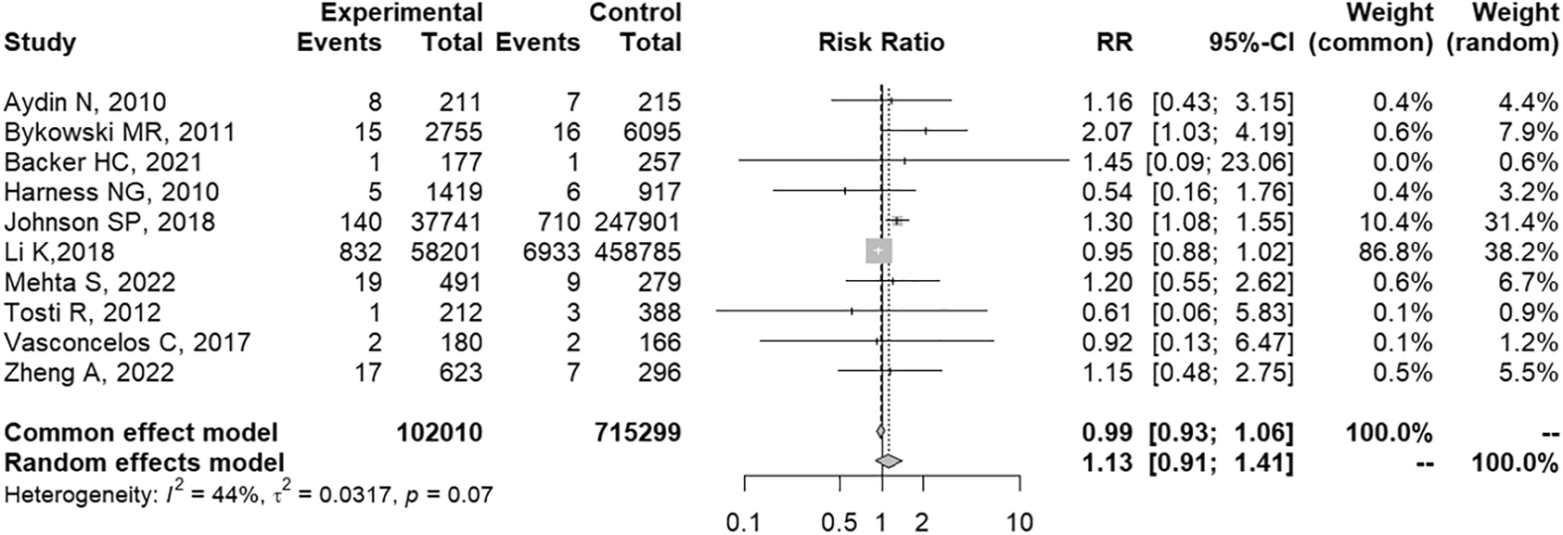
Forest plot: fixed and random effects model (R software)

Statistical heterogeneity was considered moderate (chi-squared = 16.01, degrees of freedom (df) = 9, *p* = 0.07; *I*^2^ = 44%; Tau^2^ = 0.03), so the prespecified sensitivity analysis was conducted (Fig. 5). Collected data were assessed by two independent reviewers. Studies assessing databases of medical claims were excluded from the meta-analysis, observing partial overlapping of confidence intervals and more or less similar effect estimates in the forest plot, as well as results that are consistent with those of the initial meta-analysis (Fig. 6) [6, 7]. Also, meta-analyses using fixed and random effects models had the same conclusions (Fig. 7). Therefore, despite the moderate statistical heterogeneity, the evidence found in our analysis was consistent.

**Fig. 8:**
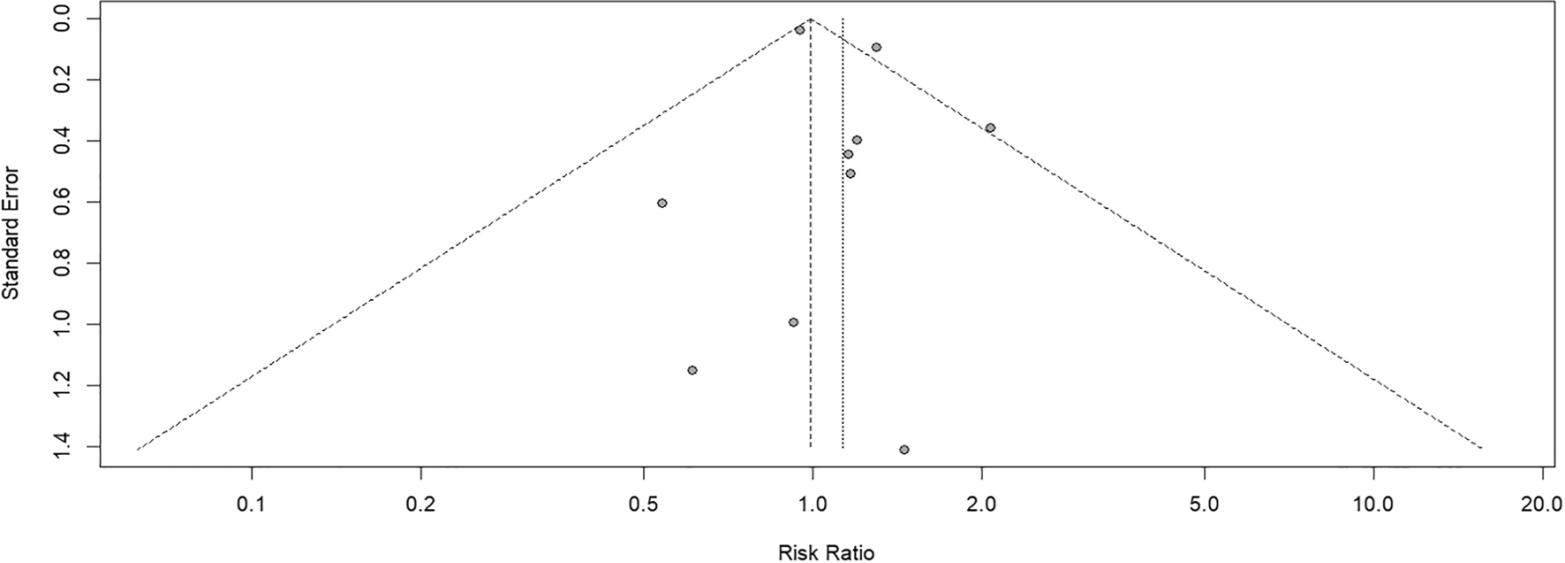
Funnel plot: risk of publication

Finally, publication bias was assessed by visual inspection of funnel plots, and no asymmetry was observed suggesting that studies with small samples and unfavorable results had not been disclosed (Fig. 8). Likewise, Eggers’s linear regression test conducted in R software confirmed this hypothesis (*t* = 0.97, df = 8, *p* = 0.36).

### Evidence of effectiveness

Most studies included cannot be considered comparable to a well-planned randomized clinical trial, indicating some problems that must be considered when interpreting the results. Also, despite a population sample of considerable size (*n* = 817,309), the number of diagnosed events (*n* = 8734), and a narrow 95% CI, the results included RR = 1. As a result, confidence in the pooled effect estimates was reduced for two reasons—methodological limitations and imprecision—and the quality of evidence was considered low in the GRADE assessment (Table 5).

**Table 5:** GRADE evidence profile

Assessment of certainty of evidence							Number of patients		Effect			
Number of studies	Study design	Risk of bias	Inconsistency	Indirection of evidence	Imprecision	Other considerations	Administration of preoperative antibiotic prophylaxis	Placebo or no drug prophylaxis	Relative risk (95% CI)	Absolute risk (95% CI)	Certainty of evidence	Importance
Surgical site infections (follow-up: from 30 to 180 days)												
10^a^	Observational study^b^	Serious^c^	Not serious^d^	Not serious^e^	Serious^f^	None	1040/102,010 (1.0%)	7694/715,299 (1.1%)	RR 1.13 (0.91–1.39)	Plus 1 per 1000 (from minus 1 to plus 4)	⨁⨁◯◯ LOW	Critical
Serious complications secondary to SSI—not measured												
–	–	–	–	–	–	–	Only 7 out of 10 studies included in this meta-analysis reported serious complications of surgical wounds, which accounted for 23% (24 out of 104) of postoperative SSI cases.^g^				–	Critical
Minor complications of surgical wounds—not measured												
–	–	–	–	–	–	–	Only 2 out of 10 studies included in the meta-analysis reported minor complications of surgical wounds. Of these, 0.89% (7 out of 780) of the patients showed these complications				–	Important
Adverse reactions and side effects to antimicrobials—not measured												
–	–	–	–	–	–	–	Only 1 out of 10 studies included in the systematic review (but not in the meta-analysis due to the number of events = 0) reported adverse reactions and side effects to antibiotics. Patients who received preoperative antibiotics had significantly more adverse reactions and side effects related to the administration of these drugs than patients who did not receive antibiotic prophylaxis (16.2% versus 5.5%; *p* = 0.029, chi-squared test). Also, 1 out of 10 patients who use antimicrobials has adverse reactions and side effects to these drugs				–	Important
Comparative costs of preoperative antibiotic prophylaxis—not measured												
–	–	–	–	–	–	–	Only 1 out of 10 studies included in the meta-analysis reported costs of preoperative antibiotic prophylaxis. For each patient, the total healthcare expenses in the first 30 days after surgery (including the date of the surgical procedure) is higher when preoperative intravenous antibiotics were administered when compared to cases that did not receive these medications (US $6070 versus US $4891, respectively; *p* < 0.001)				–	Important

*CI* confidence interval, *RR* relative risk

^a^Twelve studies were included in the systematic review; of these, ten were included in the meta-analysis and two studies found no patient with SSI in the case and/or control groups

^b^One out of ten studies included in the systematic review was a randomized, double-blind, placebo-controlled clinical trial; nine out of ten studies, on the other hand, were nonrandomized intervention studies, which together represented a weight of 96% in the meta-analysis.

^c^One out of ten studies included in the meta-analysis had a low risk of bias in the RoB 2.0 tool and nine out of ten studies had a moderate risk of bias in the ROBIS-I tool, mainly due to nonblinded measurement of the outcome by potentially biased raters

^d^The forest plot showed partial overlapping of the confidence intervals of the studies, which are more or less similar results. Also, the statistical analyses showed chi-squared = 16.01 (df = 9; p = 0.07), I2 = 44%, and Tau2 = 0.03. The prespecified sensitivity analysis showed that heterogeneity did not impact the results

^e^Although one out of ten studies excludes patients with risk factors for the occurrence of SSI, such as immunosuppression and other comorbidities, and one out of ten studies has the diagnosis of SSI inferred by the use of oral antibiotics in the postoperative period or the need for surgical re-approach, both accounted for only 10.6% weight in the meta-analysis. Likewise, these exclusions from the meta-analysis did not impact the results

^f^Despite the high number of participants (n = 817,309), the number of events (n = 8734), and narrow 95% confidence interval (0.91–1.40), the pooled effects estimates included RR = 1 and values compatible with reduction, increase, and also absence of effect, resulting in result uncertainty

^g^SSIs were considered serious when their treatment demanded new surgical procedures and/or hospitalization

#### Explanation

- Twelve studies were included in the systematic review; of these, ten were included in the meta-analysis and two studies found no patient with SSI in the case and/or control groups.
- One out of ten studies included in the systematic review was a randomized, double-blind, placebo-controlled clinical trial; nine out of ten studies, on the other hand, were nonrandomized intervention studies, which together represented a weight of 96% in the meta-analysis.
- One out of ten studies included in the meta-analysis had a low risk of bias in the RoB 2.0 tool and nine out of ten studies had a moderate risk of bias in the ROBIS-I tool, mainly due to nonblinded measurement of the outcome by potentially biased raters.
- The forest plot showed partial overlapping of the confidence intervals of the studies, which are more or less similar results. Also, the statistical analyses showed chi-squared = 16.01 (df = 9; *p* = 0.07), *I*^2^ = 44%, and Tau^2^ = 0.03. The prespecified sensitivity analysis showed that heterogeneity did not impact the results.
- Although one out of ten studies excludes patients with risk factors for the occurrence of SSI, such as immunosuppression and other comorbidities, and one out of ten studies has the diagnosis of SSI inferred by the use of oral antibiotics in the postoperative period or the need for surgical re-approach, both accounted for only 10.6% weight in the meta-analysis. Likewise, these exclusions from the meta-analysis did not impact the results.
- Despite the high number of participants (*n* = 817,309), the number of events (*n* = 8734), and narrow 95% confidence interval (0.91–1.40), the pooled effects estimates included RR = 1 and values compatible with reduction, increase, and also absence of effect, resulting in result uncertainty.
- SSIs were considered serious when their treatment demanded new surgical procedures and/or hospitalization.

### Selection bias

We believe no bias was present in the review process, as we used a comprehensive search strategy, including observational studies, as we knew beforehand the rarity of randomized, double-blind, controlled clinical trials that could answer the study question. In addition, data about the primary outcome were easily extracted from the primary studies included in our review.

## Discussion

It is unclear whether preoperative antibiotics are necessary for elective clean hand and upper limb surgeries. The dilemma lies in the potential benefits of preventing surgical site infections versus the associated risks of its use. Problems associated with the excessive use of antibiotics include an increase in bacterial resistance with consequent reduction in the overall efficacy of these drugs, risk of adverse reactions and side effects, anaphylactic shock, infections by *Clostridium difficile*, and delayed wound healing. This uncertainty puts a strain on healthcare resources in terms of personnel and finances [9, 10, 23–26].

When it comes to evaluating the effectiveness of health interventions, randomized clinical trials are considered the best study design. However, there are limited studies in scientific literature that specifically explore the use of antibiotics to prevent surgical site infections in elective clean hand and upper limb surgeries. Moreover, there are very few studies with enough sample sizes to produce reliable, statistically significant results. The fact is that, although at the top of the evidence pyramid, some questions are unlikely to be answered by authors using randomized clinical trials, and this is probably one of these questions [8].

While there are narrative reviews available on this subject, they do not make distinctions between elective and nonelective surgeries, or between procedures that solely involve soft tissue and those that include concurrent bone procedures or the placement of orthopedic implants [9, 10]. However, the decision about whether or not to prescribe prophylactic antibiotics is made every time this type of surgery is performed.

In our review, only 0.2% of patients (*n* = 24/14,255) demanded new surgical procedures and/or hospitalization related to SSI [4, 15, 16, 19–23]. Also, the prescription of preoperative prophylactic antibiotics had no impact on the incidence of SSI when compared with the administration of placebo or no prophylaxis (RR = 1.13; 95% CI 0.91–1.40; *z* = 1.1; *p* = 0.28). However, although this result is aligned with the evidence observed in the primary studies selected for this review, considering *p* ≤ 0.05 as statistically significant, no conclusion can be reached from our data meta-analysis.

Bykowski et al., in a single-center retrospective analysis of 8850 elective hand surgery cases, using a multivariate regression analysis, concluded that diabetes mellitus (OR = 2.8, 95% CI 1.2–6.5, *p* = 2 × 10^–2^), smoking (OR = 3.0, 95% CI 1.5–6.2, *p* = 3 × 10^–3^), and longer surgical time (OR = 1.02, 95% CI 1.01–1.03, *p* = 1 × 10^–4^) are positive predictors of SSI regardless of the administration of antimicrobials [16]. Shapiro et al., in a critical analysis review, found that there is a paucity of literature evaluating the use of preoperative antibiotic prophylaxis in patients with rheumatoid arthritis, those with cardiac valves, and those taking corticosteroids. There are other well-known risk factors for the occurrence of infections in general, but the literature has no study specifically assessing the effect of antimicrobials on the prevention of SSI after elective clean soft tissue surgeries of the hand and upper limb in these populations [1].

Although we do not have reasonable evidence to answer these questions, some related facts are well established in the literature; for example, the potential harmful effects of general and universal antibiotic prophylaxis which is probably minimally effective in the prevention of SSI. In this context, Sandrowski et al. observed 1.5% of adverse reactions after the preoperative single-dose administration of antibiotics to a cohort of 551 patients undergoing outpatient surgeries of the hand and upper limb [24]. According to Wachtel et al., one out of ten patients who receive antimicrobials show adverse reactions (16.2% versus 5.5%; *p* = 0.03) [22]. Likewise, a recent review described rates of up to 0.1% anaphylaxis due to the administration of cephalexin, as well as 21% diarrhea, and up to 8% infection caused by *Clostridium difficile* after the administration of clindamycin [10]. Finally, Tacconelli et al., in a systematic review of the literature and meta-analysis of total 24,230 patients, observed that exposure to antibiotics almost doubles the risk of infection by methicillin-resistant *Staphylococcus aureus* (RR = 1.8, 95% CI 1.7–1.9, *p* < 0.001) [26].

In another perspective, if prophylactic antibiotics were not routinely administered, at least US $15–30 million would be saved every year in the USA [10]. In this regard, Johnson et al. noted that total healthcare expenditures in the first 30 days after surgery are higher in cases where preoperative intravenous antibiotics are administered when compared with cases that do not receive drug prophylaxis (US $6070 versus US $4891, respectively; *p* < 0.001) [7].

## Study strengths and limitations

This study has strengths that should be highlighted. First, the PRISMA declaration guidelines were used for the development of a detailed protocol, externally reviewed and publicly registered on the international PROSPERO platform. A well-documented and sensitive search strategy enabled the retrieval of more than 1100 titles. Article selection and data extraction were performed independently by two reviewers, with disagreements arbitrated by a senior reviewer. The same procedure was used in risk of bias assessments for randomized clinical trials and nonrandomized intervention studies, as recommended by the RoB 2.0 and ROBIS-I tools, in this order. Finally, judgment and classification of the level of certainty of the evidence was performed using the structured, reproducible, and transparent approach defined in the GRADE system.

In addition to the judicious methodology, the size of the investigated population sample of 817,805 patients should also be highlighted. This large population would not probably be obtained if the studies were not combined. However, considering the general low incidence of SSI in the context of elective clean soft tissue surgeries of the hand and upper limb, a considerable population would be critical for the detection of a potentially small effect, with adequate statistical power, such as the one investigated in our review.

In contrast, our study has some limitations. First, information was retrieved from articles published in the literature and, therefore, from secondary sources. Then, data about some important developments related to antibiotic prophylaxis, for example, serious complications resulting from SSI, and adverse reactions and side effects to the use of these drugs, were not always available. Second, considering this is a systematic review, patients who met the eligibility criteria showed differences in their baseline characteristics. Also, the variables that influenced the decision of whether or not to use antibiotic prophylaxis were different among the included studies, as they depended on the surgeon’s personal experience and the tradition of the institution where the surgical procedure was performed, particularly when considering the absence of randomization and specific guidelines for antibiotic prophylaxis in elective clean soft tissue surgeries of the hand and upper limb. Finally, although the criteria for SSI diagnosis are documented and consistent with each other in the selected primary studies, no standardization was found in their definition and measurement of results, nor blinding of outcome raters regarding the group to which participants were allocated. Then, grouping of data potentially introduced confounding factors, which are inherent to systematic reviews of observational studies, although these studies remain valid and often the only feasible sources of information in the investigation of uncommon outcomes, such as SSI in this class of surgeries.

## Conclusions

### Implications for practice

Low-quality evidence suggests that there is no statistically significant difference between the use of preoperative antibiotic when compared with placebo or no drug prophylaxis for the prevention of SSI in elective clean soft tissue surgeries of the hand and upper limb. Thus, we believe that other perioperative prophylactic measures, such as hand washing, adequate skin preparation, and the use of surgical drapes and sterile technique, are more effective and less harmful than the administration of antimicrobials and therefore we discourage their use in this class of surgeries.

### Implications for research

Controlled clinical trials with appropriate randomization and blinding methods and recruitment strategies that can ensure generalization of the results obtained would be the preferred study design to assess the real efficacy of antibiotic prophylaxis in the prevention of SSI after elective clean soft tissue surgeries of the hand and upper limb. However, given the infrequency of this outcome in this class of surgeries, these clinical trials would require a population sample of thousands of participants or even more, for example, in cases including analyses of subgroups of patients with certain characteristics that make them susceptible to infections.

However, if this is not feasible, an alternative would be to conduct studies with large multicenter prospective cohorts. This would require an acceptable rate of clinically relevant SSI in terms of use of human, technical, and financial healthcare resources versus the occurrence of complications and sequelae secondary to these infections, with these prospective studies being fed until a statistically significant difference could be detected between the comparison groups. However, we may already be within an acceptable rate of SSI in this class of surgeries only with nondrug prophylactic practices generally implemented today.

Even so, these studies assessing large, multicenter prospective cohorts could support the development of a probability calculator that provides a composite measure for the risk of infection according to the patient’s health status and the type of surgical procedure, guiding the indication of preoperative antibiotic prophylaxis on a case-by-case basis and enabling an informed and shared decision between physicians and their patients.

### Supplementary Information

**Additional file 1. Table S1.** PRIMA checklist.

**Additional file 2. Table S2.** Search strategies.

**Additional file 3. Table S3.** Reports excluded.

## Data Availability

The data that support the findings of this study are available from the corresponding author upon reasonable request.

## Abbreviations

ABP: Antibiotic prophylaxis
df: Degrees of freedom
GRADE: Grading of Recommendations, Assessment, Development, and Evaluation
CI: Confidence interval
BMI: Body mass index
M-H: Mantel–Haenszel
m^2^: Square meters
kg: Kilograms
PL/∅: Placebo or no drug prophylaxis
PRISMA: Preferred Reporting Items for Systematic Reviews and Meta-analyses
PROSPERO: International Prospective Register of Systematic Reviews
RR: Relative risk
SSI: Surgical site infection
