# Supplementary material for: Preoperative antibiotic prophylaxis and the incidence of surgical site infections in elective clean soft tissue surgery of the hand and upper limb: a systematic review and meta-analysis
Source: J Orthop Traumatol. 2024 Jan 28;25:4. doi: 10.1186/s10195-024-00748-4 (PMC10822832; doi:10.1186/s10195-024-00748-4)
Supplement: Supplementary file 2 — Additional file 2. Table S2. Search strategies. [file 10195_2024_748_MOESM2_ESM.docx]

**ADDITIONAL FILES – Reports excluded**

| **Reports excluded** | | **Reasons** |
| --- | --- | --- |
| 1 | Angeles-Garay U et al (2014) Factores de riesgo relacionados con infección del sitio quirúrgico en cirugía electiva [Risk factors related to surgical site infection in elective surgery]. Cir Cir 82(1):48-62. Spanish. | Population. |
| 2 | Arulappen AL et al (2021) The Impact of Antimicrobial Stewardship Program on Injudicious Use of Cefuroxime. Front Pharmacol. DOI: 10.3389/fphar.2020.565818. | Population. |
| 3 | Bosco JA 3^rd^ et al (2010) Perioperative strategies for decreasing infection: a comprehensive evidence-based approach. The Journal of Bone & Joint Surgery 92(1):232-9. | Type of study - *Literature reviews.* |
| 4 | Eberlin KR, Ring D (2015) Infection after hand surgery. Hand Clin 31(2):355-60. DOI: 10.1016/j.hcl.2014.12.007. | Type of study - *Literature reviews.* |
| 5 | Fry DE (2017) Prevention of Infection at the Surgical Site. Surg Infect (Larchmt) 18(4):377-378. DOI: 10.1089/sur.2017.099. | Type of study - *Editorial.* |
| 6 | Hasler A et al (2021) Deep surgical site infections following double-dose perioperative antibiotic prophylaxis in adult obese orthopedic patients. Int J Infect Dis 108:537-542. DOI: 10.1016/j.ijid.2021.06.008. | Population and outcome. |
| 7 | Evans RP et al (2009) Surgical Site Infection Prevention and Control: An Emerging Paradigm. The Journal of Bone & Joint Surgery 91(6):2-9. DOI: 10.2106/JBJS.I.00549 | Type of study - *Literature reviews.* |
| 8 | Keshmiri A et al (2015) Vermeidung periprothetischer Infektionen: Nicht evidenzbasierte Maßnahmen [Prevention of periprosthetic joint infections: Not evidence-based strategies]. Orthopade 44(5):338-43. DOI: 10.1007/s00132-015-3082-3. | Type of study - *Literature reviews.* |
| 9 | Opri F et al (2022) On Behalf Of The Peri-Operative Prophylaxis In Neonatal And Paediatric Age Pop-NeoPed Study Group. Surgical Antimicrobial Prophylaxis in Patients of Neonatal and Pediatric Age Undergoing Orthopedic and Hand Surgery: A RAND/UCLA Appropriateness Method Consensus Study. Antibiotics (Basel) 11(3):289. DOI: 10.3390/antibiotics11030289. | Type of study - *Panel of experts.* |
| 10 | Osei DA, Boyer MI (2012) Preoperative antibiotic prophylaxis in the penicillin-allergic patient. J Hand Surg Am 37(12):2623-5. DOI: 10.1016/j.jhsa.2012.06.038. | Type of study - *Literature reviews.* |
| 11 | Platt AJ, Page RE (1995) Post-operative infection following hand surgery. Guidelines for antibiotic use. J Hand Surg Br 20(5):685-90. DOI: 10.1016/s0266-7681(05)80137-8. | Population. |
| 12 | Rose NE (2011) Southern California Society for Surgery of the Hand (SCSSH) selection for the Journal of Hand Surgery 2010 article of the year. J Hand Surg Am 36(9):1564. DOI: 10.1016/j.jhsa.2011.06.031. | Type of study - *Letter to the editor.* |
| 13 | Sierakowski K et al (2015) Prescribing antibiotics for hand surgery. Journal of Pharmacy Practice and Research 45 (1): 122-123. | Type of study - *Letter to the editor.* |
| 14 | Szabo RM (2010) Perioperative antibiotics for carpal tunnel surgery. J Hand Surg Am 35(1):122-4. DOI: 10.1016/j.jhsa.2009.10.023. | Type of study - *Literature reviews.* |
| 15 | Toia F et al (2012) Perioperative antibiotic prophylaxis in plastic surgery: a prospective study of 1,100 adult patients. J Plast Reconstr Aesthet Surg 65(5):601-9. DOI: 10.1016/j.bjps.2011.11.038. | Population. |
| 16 | Verma MK et al (2009) Cephalosporins in hand surgery. J Hand Surg Am 34(4):755-8. DOI: 10.1016/j.jhsa.2009.02.001. | Type of study - *Literature reviews.* |
| 17 | Werner BC et al (2018) Patient-Related Risk Factors for Infection Following Open Carpal Tunnel Release: An Analysis of Over 450,000 Medicare Patients. J Hand Surg Am 43(3):214-219. DOI: 10.1016/j.jhsa.2017.09.017. | Intervention. |
| 18 | Whittaker JP et al (2005) The role of antibiotic prophylaxis in clean incised hand injuries: a prospective randomized placebo controlled double blind trial. J Hand Surg Br 30(2):162-7. | Population. |
