# Supplementary material for: Preoperative antibiotic prophylaxis and the incidence of surgical site infections in elective clean soft tissue surgery of the hand and upper limb: a systematic review and meta-analysis
Source: J Orthop Traumatol. 2024 Jan 28;25:4. doi: 10.1186/s10195-024-00748-4 (PMC10822832; doi:10.1186/s10195-024-00748-4)
Supplement: Supplementary file 3 — Additional file 3. Table S3. Reports excluded. [file 10195_2024_748_MOESM3_ESM.docx]

**ADDITIONAL FILES – Search strategies and information sources**

| **DATABASES** | **SEARCH STRATEGIES** | **RESULTS**  **April 19^th^, 2023** |
| --- | --- | --- |
| PUBMED | ((((((Hand[MeSH Terms]) OR (Hand[Title/Abstract] OR Hands[Title/Abstract])) AND ((Upper Extremity[MeSH Terms]) OR ("Upper Extremity"[Title/Abstract] OR "Extremities, Upper"[Title/Abstract] OR "Upper Extremities"[Title/Abstract] OR "Membrum superius"[Title/Abstract] OR "Upper Limb"[Title/Abstract] OR "Limb, Upper"[Title/Abstract] OR "Limbs, Upper"[Title/Abstract] OR "Upper Limbs"[Title/Abstract] OR "Extremity, Upper"[Title/Abstract]))) OR ((Hand[MeSH Terms]) OR (Hand[Title/Abstract] OR Hands[Title/Abstract]))) AND ((General Surgery[MeSH Terms]) OR ("General Surgery"[Title/Abstract] OR "Surgery, General"[Title/Abstract] OR Surgery[Title/Abstract]))) AND (((Premedication[MeSH Terms]) OR (Premedication[Title/Abstract] OR Premedications[Title/Abstract])) OR ((Antibiotic Prophylaxis[MeSH Terms]) OR ("Antibiotic Prophylaxis"[Title/Abstract] OR "Prophylaxis, Antibiotic"[Title/Abstract] OR "Premedication, Antibiotic"[Title/Abstract] OR "Antibiotic Premedication"[Title/Abstract] OR "Antibiotic Premedications"[Title/Abstract] OR "Premedications, Antibiotic"[Title/Abstract])))) AND (((Postoperative Complications[MeSH Terms]) OR ("Postoperative Complications"[Title/Abstract] OR "Complication, Postoperative"[Title/Abstract] OR "Complications, Postoperative"[Title/Abstract] OR "Postoperative Complication"[Title/Abstract])) OR ((Surgical Wound Infection[MeSH Terms]) OR ("Surgical Wound Infection"[Title/Abstract] OR "Infections, Surgical Wound"[Title/Abstract] OR "Surgical Wound Infections"[Title/Abstract] OR "Wound Infections, Surgical"[Title/Abstract] OR "Infection, Surgical Wound"[Title/Abstract] OR "Surgical Site Infection"[Title/Abstract] OR "Infection, Surgical Site"[Title/Abstract] OR "Infections, Surgical Site"[Title/Abstract] OR "Surgical Site Infections"[Title/Abstract] OR "Wound Infection, Postoperative"[Title/Abstract] OR "Wound Infection, Surgical"[Title/Abstract] OR "Infection, Postoperative Wound"[Title/Abstract] OR "Infections, Postoperative Wound"[Title/Abstract] OR "Postoperative Wound Infections"[Title/Abstract] OR "Wound Infections, Postoperative"[Title/Abstract] OR "Postoperative Wound Infection"[Title/Abstract]))) | 148 |
| PUBMED/PMC | ((((((Hand[MeSH Terms]) OR (Hand[Title/Abstract] OR Hands[Title/Abstract])) AND ((Upper Extremity[MeSH Terms]) OR ("Upper Extremity"[Title/Abstract] OR "Extremities, Upper"[Title/Abstract] OR "Upper Extremities"[Title/Abstract] OR "Membrum superius"[Title/Abstract] OR "Upper Limb"[Title/Abstract] OR "Limb, Upper"[Title/Abstract] OR "Limbs, Upper"[Title/Abstract] OR "Upper Limbs"[Title/Abstract] OR "Extremity, Upper"[Title/Abstract]))) OR ((Hand[MeSH Terms]) OR (Hand[Title/Abstract] OR Hands[Title/Abstract]))) AND ((General Surgery[MeSH Terms]) OR ("General Surgery"[Title/Abstract] OR "Surgery, General"[Title/Abstract] OR Surgery[Title/Abstract]))) AND (((Premedication[MeSH Terms]) OR (Premedication[Title/Abstract] OR Premedications[Title/Abstract])) OR ((Antibiotic Prophylaxis[MeSH Terms]) OR ("Antibiotic Prophylaxis"[Title/Abstract] OR "Prophylaxis, Antibiotic"[Title/Abstract] OR "Premedication, Antibiotic"[Title/Abstract] OR "Antibiotic Premedication"[Title/Abstract] OR "Antibiotic Premedications"[Title/Abstract] OR "Premedications, Antibiotic"[Title/Abstract])))) AND (((Postoperative Complications[MeSH Terms]) OR ("Postoperative Complications"[Title/Abstract] OR "Complication, Postoperative"[Title/Abstract] OR "Complications, Postoperative"[Title/Abstract] OR "Postoperative Complication"[Title/Abstract])) OR ((Surgical Wound Infection[MeSH Terms]) OR ("Surgical Wound Infection"[Title/Abstract] OR "Infections, Surgical Wound"[Title/Abstract] OR "Surgical Wound Infections"[Title/Abstract] OR "Wound Infections, Surgical"[Title/Abstract] OR "Infection, Surgical Wound"[Title/Abstract] OR "Surgical Site Infection"[Title/Abstract] OR "Infection, Surgical Site"[Title/Abstract] OR "Infections, Surgical Site"[Title/Abstract] OR "Surgical Site Infections"[Title/Abstract] OR "Wound Infection, Postoperative"[Title/Abstract] OR "Wound Infection, Surgical"[Title/Abstract] OR "Infection, Postoperative Wound"[Title/Abstract] OR "Infections, Postoperative Wound"[Title/Abstract] OR "Postoperative Wound Infections"[Title/Abstract] OR "Wound Infections, Postoperative"[Title/Abstract] OR "Postoperative Wound Infection"[Title/Abstract]))) | 02 |
| BVS / BIREME  MEDLINE (176)  IBECS (6)  LILACS (4)  WPRIM (3)  BDENF (1)  BINACIS (1)  CUMED (1) | (((hand OR hands) OR ((hand OR hands) AND ("Upper Extremity" OR "Extremities, Upper" OR "Upper Extremities" OR "Membrum superius" OR "Upper Limb" OR "Limb, Upper" OR "Limbs, Upper" OR "Upper Limbs" OR "Extremity, Upper"))) AND (("General Surgery" OR "Surgery, General" OR surgery))) AND (("Antibiotic Prophylaxis" OR "Prophylaxis, Antibiotic" OR "Premedication, Antibiotic" OR "Antibiotic Premedication" OR "Antibiotic Premedications" OR "Premedications, Antibiotic") OR (premedication OR premedications)) AND (("Postoperative Complications" OR "Complication, Postoperative" OR "Complications, Postoperative" OR "Postoperative Complication") OR ("Surgical Wound Infection" OR "Infections, Surgical Wound" OR "Surgical Wound Infections" OR "Wound Infections, Surgical" OR "Infection, Surgical Wound" OR "Surgical Site Infection" OR "Infection, Surgical Site" OR "Infections, Surgical Site" OR "Surgical Site Infections" OR "Wound Infection, Postoperative" OR "Wound Infection, Surgical" OR "Infection, Postoperative Wound" OR "Infections, Postoperative Wound" OR "Postoperative Wound Infections" OR "Wound Infections, Postoperative" OR "Postoperative Wound Infection")) | 190 |
| SCOPUS | ( ( ( ( TITLE-ABS-KEY ( hand OR hands ) AND TITLE-ABS-KEY ( "Upper Extremity" OR "Extremities, Upper" OR "Upper Extremities" OR "Membrum superius" OR "Upper Limb" OR "Limb, Upper" OR "Limbs, Upper" OR "Upper Limbs" OR "Extremity, Upper" ) ) ) OR ( TITLE-ABS-KEY ( hand OR hands ) ) ) AND ( TITLE-ABS-KEY ( "General Surgery" OR "Surgery, General" OR surgery ) ) ) AND ( ( TITLE-ABS-KEY ( "Antibiotic Prophylaxis" OR "Prophylaxis, Antibiotic" OR "Premedication, Antibiotic" OR "Antibiotic Premedication" OR "Antibiotic Premedications" OR "Premedications, Antibiotic" ) OR TITLE-ABS-KEY ( premedication OR premedications ) ) ) AND ( ( TITLE-ABS-KEY ( "Postoperative Complications" OR "Complication, Postoperative" OR "Complications, Postoperative" OR "Postoperative Complication" ) OR TITLE-ABS-KEY ( "Surgical Wound Infection" OR "Infections, Surgical Wound" OR "Surgical Wound Infections" OR "Wound Infections, Surgical" OR "Infection, Surgical Wound" OR "Surgical Site Infection" OR "Infection, Surgical Site" OR "Infections, Surgical Site" OR "Surgical Site Infections" OR "Wound Infection, Postoperative" OR "Wound Infection, Surgical" OR "Infection, Postoperative Wound" OR "Infections, Postoperative Wound" OR "Postoperative Wound Infections" OR "Wound Infections, Postoperative" OR "Postoperative Wound Infection" ) ) ) | 285 |
| WEB OF SCIENCE | (Hand OR Hands (Topic) and "Upper Extremity" OR "Extremities, Upper" OR "Upper Extremities" OR "Membrum superius" OR "Upper Limb" OR "Limb, Upper" OR "Limbs, Upper" OR "Upper Limbs" OR "Extremity, Upper" (Topic) and Preprint Citation Index (Exclude – Database) OR Hand OR Hands (Topic) and Preprint Citation Index (Exclude – Database)) AND "General Surgery" OR "Surgery, General" OR Surgery (Topic) Preprint Citation Index (Exclude – Database) AND ("Antibiotic Prophylaxis" OR "Prophylaxis, Antibiotic" OR "Premedication, Antibiotic" OR "Antibiotic Premedication" OR "Antibiotic Premedications" OR "Premedications, Antibiotic" (Topic) or Premedication OR Premedications (Topic) and and Preprint Citation Index (Exclude – Database)) AND ("Postoperative Complications" OR "Complication, Postoperative" OR "Complications, Postoperative" OR "Postoperative Complication" (Topic) or "Surgical Wound Infection" OR "Infections, Surgical Wound" OR "Surgical Wound Infections" OR "Wound Infections, Surgical" OR "Infection, Surgical Wound" OR "Surgical Site Infection" OR "Infection, Surgical Site" OR "Infections, Surgical Site" OR "Surgical Site Infections" OR "Wound Infection, Postoperative" OR "Wound Infection, Surgical" OR "Infection, Postoperative Wound" OR "Infections, Postoperative Wound" OR "Postoperative Wound Infections" OR "Wound Infections, Postoperative" OR "Postoperative Wound Infection" (Topic) and Preprint Citation Index (Exclude – Database)  [*https://www.webofscience.com/wos/alldb/summary/4d59637d-3c4e-4605-a79f-08f211a56559-836d5150/author-ascending/1*](https://www.webofscience.com/wos/alldb/summary/4d59637d-3c4e-4605-a79f-08f211a56559-836d5150/author-ascending/1) | 195 |
| EMBASE | ('postoperative complication'/syn OR 'surgical infection'/syn) AND ('antibiotic prophylaxis'/syn OR 'premedication'/syn) AND ('general surgery'/syn OR 'surgery'/syn) AND ('hand'/syn OR ('hand'/syn AND 'upper limb'/syn)) AND ([embase]/lim NOT ([embase]/lim AND [medline]/lim) OR ([medline]/lim NOT ([embase]/lim AND [medline]/lim) NOT ([embase classic]/lim AND [medline]/lim))) | 291 |
| COCHRANE LIBRARY | (MeSH descriptor: [Antibiotic Prophylaxis] explode all trees OR ("Antibiotic Prophylaxis" OR "Prophylaxis, Antibiotic" OR "Premedication, Antibiotic" OR "Antibiotic Premedication" OR "Antibiotic Premedications" OR "Premedications, Antibiotic"):ti,ab,kw OR MeSH descriptor: [Premedication] explode all trees OR (Premedication OR Premedications):ti,ab,kw) AND (MeSH descriptor: [Hand] explode all trees OR (Hand OR Hands):ti,ab,kw AND MeSH descriptor: [Upper Extremity] explode all trees OR ("Upper Extremity" OR "Extremities, Upper" OR "Upper Extremities" OR "Membrum superius" OR "Upper Limb" OR "Limb, Upper" OR "Limbs, Upper" OR "Upper Limbs" OR "Extremity, Upper"):ti,ab,kw) OR MeSH descriptor: [Hand] explode all trees OR (Hand OR Hands):ti,ab,kw) AND (MeSH descriptor: [General Surgery] explode all trees OR ("General Surgery" OR "Surgery, General" OR Surgery):ti,ab,kw) AND ( MeSH descriptor: [Postoperative Complications] explode all trees OR ("Postoperative Complications" OR "Complication, Postoperative" OR "Complications, Postoperative" OR "Postoperative Complication"):ti,ab,kw OR MeSH descriptor: [Surgical Wound Infection] explode all trees OR ("Surgical Wound Infection" OR "Infections, Surgical Wound" OR "Surgical Wound Infections" OR "Wound Infections, Surgical" OR "Infection, Surgical Wound" OR "Surgical Site Infection" OR "Infection, Surgical Site" OR "Infections, Surgical Site" OR "Surgical Site Infections" OR "Wound Infection, Postoperative" OR "Wound Infection, Surgical" OR "Infection, Postoperative Wound" OR "Infections, Postoperative Wound" OR "Postoperative Wound Infections" OR "Wound Infections, Postoperative" OR "Postoperative Wound Infection"):ti,ab,kw) | 64 |
| **TOTAL 1.175** | | |
